# *Leptospira interrogans*  and *Leptospira kirschneri* are the dominant *Leptospira* species causing human leptospirosis in Central Malaysia

**DOI:** 10.1371/journal.pntd.0008197

**Published:** 2020-03-23

**Authors:** Noraini Philip, Norliza Bahtiar Affendy, Siti Nur Alia Ramli, Muhamad Arif, Pappitha Raja, Elanngovan Nagandran, Pukunan Renganathan, Niazlin Mohd Taib, Siti Norbaya Masri, Muhamad Yazli Yuhana, Leslie Thian Lung Than, Mithra Seganathirajah, Cyrille Goarant, Marga G. A. Goris, Zamberi Sekawi, Vasantha Kumari Neela

**Affiliations:** 1 Department of Medical Microbiology and Parasitology, Faculty of Medicine and Health Sciences, Universiti Putra Malaysia, Serdang, Selangor, Malaysia; 2 Center of Excellence in Life Sciences, Bharathidasan University, Tiruchirappalli, India; 3 School of Medicine, Dentistry and Biomedical Sciences, Queens University, Belfast, Northern Ireland, United Kingdom; 4 Clinical Research Centre Unit, Tengku Ampuan Rahimah Hospital, Ministry of Health Malaysia, Klang, Selangor, Malaysia; 5 Infectious Diseases Unit, Internal Medicine Department, Universiti Teknologi MARA, Sungai Buloh, Selangor, Malaysia; 6 General Medicine, Hospital Serdang, Ministry of Health Malaysia, Jalan Puchong, Kajang, Selangor, Malaysia; 7 Institut Pasteur de Noume´a, Leptospirosis Research and Expertise Unit, Noume´a, New Caledonia; 8 OIE and National Collaborating Centre for Reference and Research on Leptospirosis Academic Medical Center, Department of Medical Microbiology, University of Amsterdam, Amsterdam, the Netherlands; University of Minnesota, UNITED STATES

## Abstract

**Background:**

Leptospirosis, commonly known as rat-urine disease, is a global but endemic zoonotic disease in the tropics. Despite the historical report of leptospirosis in Malaysia, the information on human-infecting species is limited. Determining the circulating species is important to understand its epidemiology, thereby to strategize appropriate control measures through public health interventions, diagnostics, therapeutics and vaccine development.

**Methodology/Principle findings:**

We investigated the human-infecting *Leptospira* species in blood and serum samples collected from clinically suspected leptospirosis patients admitted to three tertiary care hospitals in Malaysia. From a total of 165 patients, 92 (56%) were confirmed cases of leptospirosis through Microscopic Agglutination Test (MAT) (n = 43; 47%), Polymerase Chain Reaction (PCR) (n = 63; 68%) or both MAT and PCR (n = 14; 15%). The infecting *Leptospira* spp., determined by partial 16S rDNA (*rrs*) gene sequencing revealed two pathogenic species namely *Leptospira interrogans* (n = 44, 70%) and *Leptospira kirschneri* (n = 17, 27%) and one intermediate species *Leptospira wolffii* (n = 2, 3%). Multilocus sequence typing (MLST) identified an isolate of *L*. *interrogans* as a novel sequence type (ST 265), suggesting that this human-infecting strain has a unique genetic profile different from similar species isolated from rodents so far.

**Conclusions/Significance:**

*Leptospira interrogans* and *Leptospira kirschneri* were identified as the dominant *Leptospira* species causing human leptospirosis in Central Malaysia. The existence of novel clinically important ST 265 (infecting human), that is different from rodent *L*. *interrogans* strains cautions reservoir(s) of these *Leptospira* lineages are yet to be identified.

## Author summary

Leptospirosis is a zoonotic disease caused by pathogenic *Leptospira* species. To date, the genus *Leptospira* contains 64 species isolated from human, animals and environments. Identification of *Leptospira* species causing human leptospirosis is important to decipher the epidemiology and transmission pattern of this disease. In our study, we identified *Leptospira interrogans*, *Leptospira kirschneri*, and *Leptospira wolffii* as the human-infecting *Leptospira* species in Malaysia. These three species have been isolated from rodents and environment in Malaysia earlier, however, these novel isolates are genotypically distinct. The identification of novel clinically important *Leptospira interrogans* strains in the present study underscores the importance of characterizing more human-infecting strains and search for the potential animal reservoirs.

## Introduction

Leptospirosis is a worldwide, life-threatening zoonotic disease that accounts for an estimated 58,900 deaths and more than one million cases annually [1,2]. The disease is caused by pathogenic *Leptospira* species which represent over 250 different serovars [3,4]. Although leptospirosis is globally distributed, the tropical regions of South and Southeast Asia, Africa, Western Pacific, and Central and South America harbour the highest estimated burden of this disease [2]. Leptospires are maintained by chronic carrier hosts, mainly rodents, in their renal tubules and excreted into the environment through their urine. Infection in humans results from direct contact with the infected reservoir animals or indirect exposure to the contaminated environments.

Human leptospirosis is typically characterized by a range of clinical manifestations, from mild asymptomatic infections to severe, life-threatening illness. The clinical signs and symptoms resemble several other febrile illnesses like dengue and malaria, leading to frequent misdiagnosis. Hence, leptospirosis is under-reported, and the actual public health impact is difficult to determine [1,5]. The definitive diagnosis of leptospirosis is based on several methods: isolation of the organism through culture, detection of organism’s DNA by Polymerase Chain Reaction (PCR) or detection of antibodies by Microscopic Agglutination Test (MAT). Culture is advantageous in term of information and material, however, the slow growth, low positivity, long incubation period and low recovery rate hinder the usefulness of this method for early diagnosis. MAT is the gold standard for serological diagnosis of leptospirosis yet has low sensitivity in the early course of infection, as the level of detectable IgM and IgG antibodies are low during this phase [6]. Furthermore, it requires a high level of technical expertise and the maintenance of a large panel of live pathogenic *Leptospira* representing both international and local serovars. PCR though expensive for resource-limited countries and requiring technical expertise to interpret, remains the most sensitive test for early detection of the pathogen in clinical samples. Molecular approaches do not only allow the detection of microorganism of interest but also enable the determination of species or strain of the infecting agent. To date, the genus *Leptospira* contains 64 species comprising pathogenic, intermediate and saprophytes [7–9]. Identification of infecting *Leptospira* species mainly in clinical specimens is important to determine the clinical significance, the probable source of infection and to distinguish sporadic cases from possible outbreaks. MAT can infer the serogroup of *Leptospira*, but not determine the infecting *Leptospira* species [10]. Furthermore, the concept of “serogroup” has no official taxonomic status and fails to define epidemiologically important strains or isolates [11].

In Malaysia, leptospirosis is a historical endemic disease. Cases have been reported since the 1920s among the civilians and military troops [12]. However, only after its declaration as a notifiable disease in 2010, the approximate morbidity and mortality were determined based on the number of reported cases. Since then, at least 41,736 cases (probable and confirmed) with 502 deaths have been recorded, corresponding to an average incidence of 16 cases per 100,000 population annually (data from Ministry of Health, Malaysia). In Malaysia, the patients go to primary health-care centers. Leptospirosis suspected patients are then referred to the tertiary care hospital. As a routine diagnosis, all clinically suspected leptospirosis cases are subjected to serology based on a rapid test or ELISA. Positive ones are submitted for MAT and PCR (only upon request) to the reference laboratories. Paired serum samples are occasionally available for confirmation. Hence, the knowledge of the human-infecting *Leptospira* species in Malaysia is not well determined. Management of leptospirosis is not limited to the hospital. The zoonotic nature of the illness emphasizes the importance of One Health approaches in understanding the distribution and the transmission sources for effective management of the disease. Understanding the interconnection between the animals, human and environment is important in achieving optimal health outcomes. In an earlier investigation, we characterized *Leptospira* species isolated from rodents and small mammals captured in leptospirosis hot spots and outbreak areas [13]. The present study was undertaken to identify and characterize the human-infecting *Leptospira* species in patients with leptospirosis in the two states in Central Malaysia (Selangor and Perak). Selangor had incidence rates of 14.24 and 11.30 while Perak reported 13.60 and 11.41 for the years 2016 and 2017 respectively. This information is vital to understand the potential source and most importantly for surveillance and effective control measures.

## Methods

### Ethics approval

The ethical approval for this study was obtained from the Medical Research and Ethical Committee (MREC), Ministry of Health Malaysia (NMRR-15-2148-27536). Written informed consent was obtained from all patients who participated in the study.

### Patient recruitment and sampling

We performed a prospective multi-centric study to identify the human-infecting *Leptospira* species in Malaysia. Patients clinically suspected for leptospirosis were identified by the attending physician based on the guidelines from the Ministry of Health, Malaysia [14]. These guidelines define clinical case as a patient with acute febrile illness with history of exposure to water and/or environment possibly contaminated with infected animal urine with any of the following symptoms such as headache, myalgia, arthralgia, meningeal irritation, jaundice, conjunctival suffusion, skin rash, anuria or oliguria, cardiac arrhythmia or failure, haemorrhages in intestines or lungs and gastrointestinal symptoms. Patients were recruited from two states in Central Malaysia (Selangor and Perak). The study was conducted in three hospitals (Hospital Serdang and Hospital Tengku Ampuan Rahimah (HTAR) in Selangor state and Hospital Teluk Intan in the Perak state) from January 2016 to December 2017. Blood specimens were collected upon admission from all participants using plain tube and EDTA blood tube for serology and PCR tests respectively. Upon availability, blood specimen for serological testing was also collected from the recruited patients on discharge and two weeks after discharge. Blood collected in EDTA tube was stored in -40°C freezer until DNA extraction. Socio-demography, clinical data and possible risk exposures for all participated patients were recorded using a standardized interviewer-administered questionnaire (S1 File).

### Serology and molecular characterization

To determine the seropositivity, all serum samples were subjected to MAT as described earlier [15]. For molecular detection and identification, a 242 bp *lipL32* fragment (primers: LipL32-45F: AAGCATTACCGCTTGTGGTG, LipL32-286R: GAACTC CCATTTCAGCGA TT, probe: LipL32-189P: FAM-5′-AAAGCCAGGACAAGCGCCG-3′-BHQ1) [16] which is only present in pathogenic *Leptospir*a and the 547 bp fragment of 16S rDNA (*rrs*) nested PCR assays (outer primers: rrsouter-F: CTCAGAACTAACGCTGGCGGCGCG, rrs-outer-R: GGTTCGTTACTGAGGGTTAAAACCCCC, inner primers: rrs-inner-F: CTGGCGGCGCGTCTTA, rrs-inner-R: GTTTTCACACCTGACTTACA) [17] were performed in all samples. For 16S rDNA, all nested PCR products were purified and sequenced (MyTACG Bioscience Enterprise, Malaysia). All sequences were edited and trimmed by using Mega 7.0 software (DNASTAR Inc., Wisconsin, USA) and compared against the GenBank database using BLAST to identify the species [18].

Multilocus sequence typing (MLST scheme 1) was performed based on the extended, nested MLST targeting seven loci (*glmU*, *pntA*, *sucA*, *tpiA*, *pfkB*, *mreA*, *caiB*) of seven pathogenic *Leptospira* species (*L*. *alexanderi*, *L*. *borgpetersenii*, *L*. *interrogans*, *L*. *kirschneri*, *L*. *noguchi*, *L*. *santarosai*, *L*. *weilii*) [19,20]. For sequence type definition, allelic profiles were analysed through the *Leptospira* MLST database (http://pubmlst.org/leptospira). All primers used in MLST are listed in Table 1.

**Table 1:** List of primers used in this study.

No	Gene	Primers/Probe	Sequences (5’-3’)	Size (bp)	References
1.	*glmU*	glmU-F_M_	AGGATAAGGTCGCTGTGGTA	650	[20]
		glmU-R_M_	AGTTTTTTTCCGGAGTTTCT		
2.	*pntA*	pntA-F_M_	TAGGAAARATGAAACCRGGAAC	621	
		pntA-R_M_	AAGAAGCAAGATCCACAAYTAC		
3.	*sucA*	sucA-F_M_	TCATTCCACTTYTAGATACGAT	640	
		sucA-R_M_	TCTTTTTTGAATTTTTGACG		
4.	*tpiA*	tpiA-F_M_	TTGCAGGAAACTGGAAAATGAAT	639	
		tpiA-R_M_	GTTTTACRGAACCHCCGTAGAGAAT		
5.	*pfkB*	pfkB-F_M_	CGGAGAGTTTTATAARAAGGACAT	588	
		pfkB-R_M_	AGAACACCCGCCGCAAAACAAT		
6.	*mreA*	mreA-F_M_	GGCTCGCTCTYGACGGAAA	719	
		mreA-R_M_	TCCRTAACTCATAAAMGACAAAGG		
7.	*caiB*	caiB-F	CAACTTGCGGAYATAGGAGGAG	650	
		caiB-R	ATTATGTTCCCCGTGAYTCG		
8.	*glmU*	1-glmU-2F_M13	TGTAAAACGACGGCCAGT CGYATG AAAACGGATCAG	598	[19]
		1-glmU-2R_M13	CAGGAAACAGCTATGACC GGAAGRTARTATT CDCCCTG		
9.	*pntA*	2-pntA-2F_M13	TGTAAAACGACGGCCAGT ATTTAT YTVGGRATGTTYCA	607	
		2-pntA-2R_M13	CAGGAAACAGCTATGACC GATTTCATRTTA TCYACRAT		
10.	*sucA*	3-sucA-2F_M13	TGTAAAACGACGGCCAGT GCSGGTRATCATCWBATGG	552	
		3-sucA-2R_M13	CAGGAAACAGCTATGACC GRAAWCCYTTYGCAAGATC		
11.	*tpiA*	4-tpiA-2F_M13	TGTAAAACGACGGCCAGT ATTTCYTTACGAAT RAAAGARTG	555	
		4-tpiA-2R_M13	CAGGAAACAGCTATGACC CMCATTCGATYMRAGAAAA		
12.	*pfkB*	5-pfkB-2F_M13	TGTAAAACGACGGCCAGT GTYGTATCGATC GSYTTC	540	
		5-pfkB-2R_M13	CAGGAAACAGCTATGACC YYCCSGAAGAYAAS GGWCAT		
13.	*mreA*	6-mreA-2F_M13	TGTAAAACGACGGCCAGT CRRGAAGYRGTGGATCAGG	568	
		6-mreA-2R_M13	CAGGAAACAGCTATGACC CKATCCTTACTYTCRTARCT		
14.	*caiB*	7-caiB-2F_M13	TGTAAAACGACGGCCAGT CTTKCTTCRATYTTGGCG	589	
		7-caiB-2R_M13	CAGGAAACAGCTATGACC AMCGATATGTWAY MGGRGTT		

### Statistical analysis

The results were analysed using frequency and one-sample Test and expressed through tables and summary measures. Categorical data were presented as frequency counts (N, %) while continuous variables were presented as mean **±** standard deviation. Cross-Tabulation with Chi-Square tests was used to determine if the clinical presentations at admission are statistically significant in leptospirosis patients. Chi-Square test was also used to determine the association between *Leptospira* species and collection sites. All statistical analyses were performed using IBM SPPS Statistics 22.

## Results

### Clinical signs, possible risk exposures and laboratory confirmation

**Fig 1:**
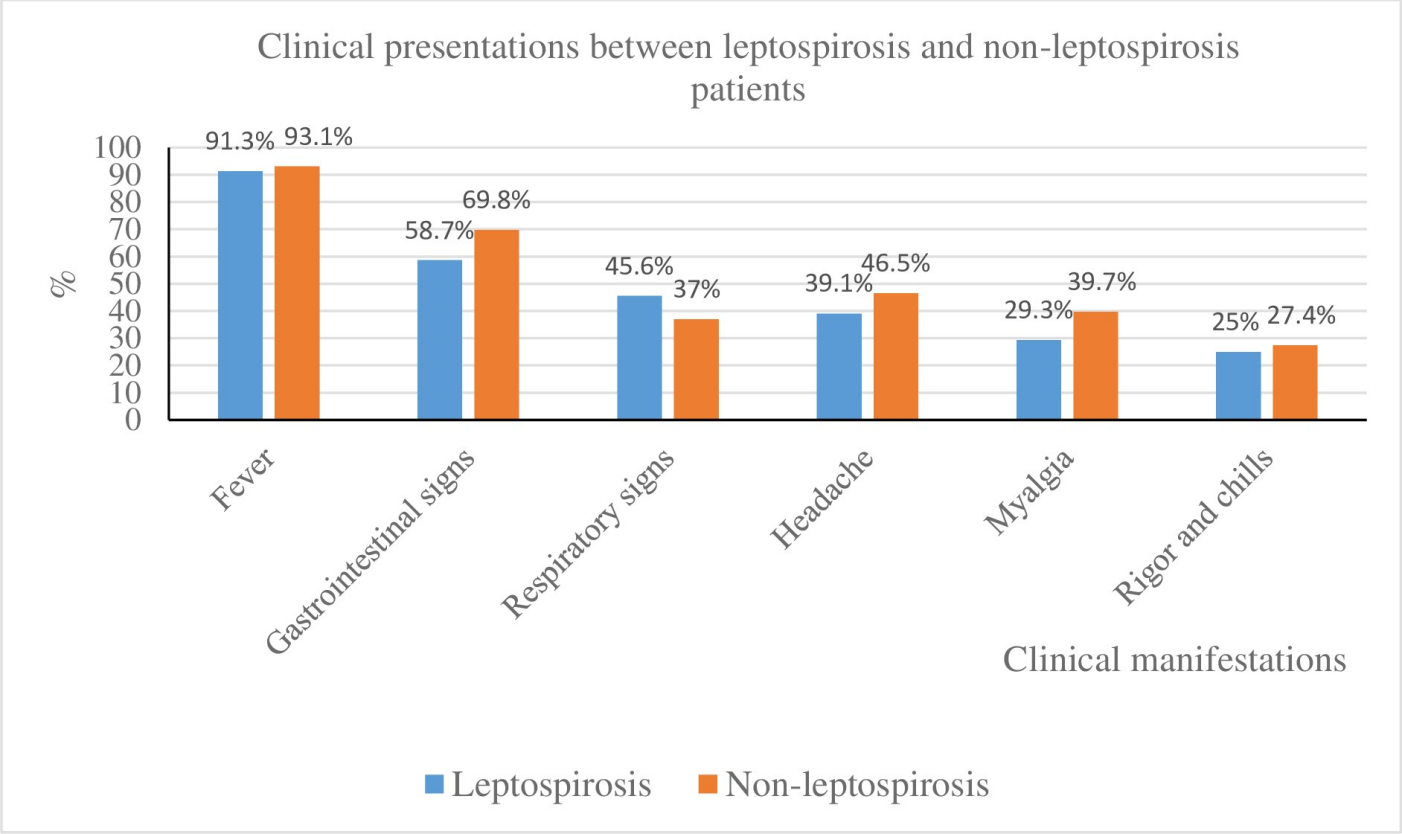
Comparison of clinical features in leptospirosis and non-leptospirosis patients.

A total of 165 patients clinically suspected for leptospirosis (Serdang Hospital (n = 59), HTAR (n = 16), Teluk Intan Hospital (n = 90)) were enrolled in this study. Among the 165, paired sera were available for 63 patients (38.1%) while the remaining patients rejected the invitation for a follow up after discharge or could not be contacted. Only samples positive by PCR or MAT (single titer ≥1:400 or paired serum with four-fold or greater rise) or both were recorded as confirmed leptospirosis [6]. Overall 92 (56%) patients were confirmed positive for leptospirosis comprising MAT (n = 43; 47%), PCR (n = 63; 68%) or MAT and PCR (n = 14; 15%). The age of infected patients ranged from 14 to 77 with a mean of 42.23 ± 18.07 years (median value = 38 ± 18.07). More than half of these patients were males (n = 60; 65%), as shown in Table 2. The mean length between the onset of symptoms and hospital admission was found to be 5.02 ± 3.5 days (range 1 to 15 days). About 54.3% of the patients reported exposure to rats, 34.8% were involved with outdoor activities before the illness, 9.8% had contact with pets and 1.09% had exposure to floodwater. For those clinically suspected for leptospirosis, the main clinical signs and symptoms at the time of admission included fever (91.3%), gastrointestinal problems (58.7%), respiratory problems (45.6%), headache (39.1%), myalgia (29.3%) and, rigor and chills (25%), Fig 1. However, none of these characteristics showed a significant difference between the laboratory confirmed-leptospirosis and non-leptospirosis patients (MAT and PCR negative), p-value>0.001. Of the 92 confirmed leptospirosis patients, a total of 34 were identified as severe cases based on the criteria described elsewhere [21]. The criteria for severe cases included any organ dysfunction, jaundice or total bilirubin >3.5mg/dl or high ALT>120IU (alanine aminotransferase) or pulmonary dysfunction or hemoptysis or requiring mechanical ventilation support or renal dysfunction or oliguria, abnormal creatinine level (30-100umol/L). Among the 34 severe cases, 11 were fatal, of which 10 showed acute kidney injury (AKI).

**Table 2:** Demography and risk exposures of patients with confirmed leptospirosis.

Baseline characteristics	Mean ±SD
Age (years)	42.23 ± 18.07
Onset of symptoms to admission (days)	5.02 ± 3.5
Gender	n (%)
Male	60 (65%)
Female	32 (35%)
**Risk exposures**	**n (%)**
Contact with rats	50 (54.3%)
Outdoors activities (jungle trekking and recreational water activities)	33 (34.8%)
Contact with pets (cats/dogs)	9 (9.8%)
Exposure to flood	1(1.09%)

All leptospirosis patients were also tested for co-infection with other diseases with similar clinical presentations. Among these patients, seven (7.6%) and one patient (1.1%) had co-infection with dengue and scrub typhus respectively.

### Molecular characterization of infecting *Leptospira* spp.

Among the 165 clinically suspected leptospirosis patients, *lipL32* was detected in 47 (28.5%) and *rrs* gene in 63 (38.1%) patients. All *lipL32*-PCR positive samples were positive by the *rrs* PCR. The blood and serum samples that were positive by *lipL32* showed Ct values between 28.96 and 39.67.

The BLAST analysis of partial 16S rDNA (*rrs*) gene sequencing revealed two pathogenic *Leptospira* species with 100% identity, which are *L*. *interrogans* (n = 44, 70%) and *L*. *kirschneri* (n = 17, 27%), and one intermediate species *L*. *wolffii* (n = 2, 3%). The identity of the isolates and the accession numbers of the 16S rDNA for all isolates are listed in Supplementary Table 1 (S2 File). In Selangor hospitals (Hospital Serdang and HTAR), *L*. *kirschneri* was found to be more prevalent while *L*. *interrogans* dominated in Hospital Teluk Intan (Perak state), p-value<0.001 (Table 3).

**Table 3:** Number of *Leptospira* spp. from the three hospitals in Selangor and Perak states, Malaysia.

Species	Selangor	Perak	Overall
*L*. *interrogans*	12/28 (43%)	32/35 (91%)	44/63 (70%)
*L*. *kirschneri*	14/28 (50%)	3/35 (9%)	17/63 (27%)
*L*. *wolffii*	2/28 (7%)	-	2/63 (3%)

MLST was attempted in all PCR positive cases (n = 63). However, only those samples with successful amplification of three or more alleles were included for the analysis. A total of nine blood samples showed amplification for at least three alleles, however only one gave a complete MLST profile. For the sample that showed complete MLST profile, the combination of alleles did not match a known sequence type (ST) in the MLST database for *Leptospira* species, hence a new ST was created with the help of the MLST curator (www.pubmlst.net) and identified the isolate as ST 265 *Leptospira interrogans*. The remaining eight samples amplified at least three loci (Table 4). The loci that were most frequently amplified were *glmU* and *pntA* (n = 9) followed by *mreA* (n = 5), *sucA* (n = 4), *pfkB*, *tpiA* and *caiB* (n = 3). We tried to arrange the allele pattern for the *Leptospira* MLST genes detected in human (present study) and rodents from our previous study [13]. This was done to see whether they share similar alleles in order to determine the epidemiological link as the ST was not available for the human infecting strains to compare with the animal strains. From the MLST dataset (Table 4) no link could be established between the human-infecting strains and rats strains (ST 50, ST 238, ST 243 *L*. *interrogans* and ST 110 *L*. *kirschneri*). None of the seven genes in the human and animal showed similar alleles except for *pntA (L*. *interrogans)*, *sucA (L*. *interrogans and L*. *kirschneri)*, *tpiA (L*. *interrogans)*, *mreA (L*. *interrogans)*, however, the allele combinations were different.

**Table 4:** Allelic profiles of the nine human samples, four animals and two *Leptospira* strains from MLST databases.

Isolate ID	Host	Species	MLST								References
			ST	*glmU*	*pntA*	*sucA*	*tpiA*	*pfkB*	*mreA*	*caiB*	
SP15	Human	*L*. *interrogans*	*265*	9	1	2	2	106	2	3	This study
SP26	Human	*L*. *interrogans*		9	1	-	2	-	2	-	This study
SP27	Human	*L*. *interrogans*		9	1	-	2	-	2	-	This study
SP29	Human	*L*. *interrogans*		9	1	2	-	-	2	8	This study
SP32	Human	*L*. *interrogans*		9	1	-	-	-	2	-	This study
3705	Human	***L*. *interrogans***	***58***	**9**	**1**	**2**	**4**	**13**	**9**	**3**	**[20, 22]**
SC244	Rat	***L*. *interrogans***	***50***	**6**	**8**	**2**	**2**	**9**	**7**	**5**	**[13]**
WRG-L358	Rat	***L*. *interrogans***	***238***	**1**	**1**	**2**	**4**	**1**	**2**	**6**	**[13]**
WRG-L349	Rat	***L*. *interrogans***	***243***	**1**	**1**	**1**	**1**	**8**	**6**	**6**	**[13]**
SP14	Human	*L*. *kirschneri*		16	34	-	-	-	-	-	This study
SP28	Human	*L*. *kirschneri*		16	34	13	-	-	-	-	This study
SP44	Human	*L*. *kirschneri*		16	34	-	-	76	-	-	This study
SP47	Human	*L*. *kirschneri*		16	34	13	-	31	-	24	This study
Butembo	Human	***L*. *kirschneri***	***69***	**16**	**20**	**13**	**22**	**31**	**18**	**23**	**[20, 22]**
SGS118	Rat	***L*. *kirschneri***	***110***	**19**	**20**	**13**	**22**	**31**	**18**	**23**	**[13]**

## Discussion

Leptospirosis remains a burdensome and challenging disease. The vast variations in clinical presentations hinder the early accurate diagnosis, hence laboratory confirmation is critically needed to support the manifested signs and symptoms for initiating the treatment. In the present study, 92/165 patients with clinical manifestations suggesting leptospirosis were confirmed through MAT and/or PCR. The mean age of the leptospirosis confirmed patients was 42.23 ± 18.07, which is in agreement with several other studies that ranged between 38.9–45 years [23–27]. About two thirds of the patients (n = 60; 65%) were males as observed in most studies [24–26, 28, 29]. Although leptospirosis can affect all age groups and both genders, active involvement in outdoor activities increases its incidence in adults and males. More than half (54.3%) of the patients had a history of contact with rats indicating that exposure to rats increases their risk of getting the disease [30]. Secondly, we found outdoor activities as the major risk factor which is in agreement with our earlier finding and also elsewhere [30–33]. While it is true that flood increases the number of leptospirosis cases, in the present study, only one out of the 92 confirmed cases reported floodwater exposure. Hence, we were not able to associate flood with leptospirosis for this current study. Fever, respiratory problems, headache, myalgia, chills and rigors were observed in most confirmed leptospirosis patients which agree with several other studies reported earlier [5, 23–29, 34, 35]. However, in addition to the traditional symptoms mentioned above, we observed gastrointestinal problems such as vomiting, abdominal pain, nausea and diarrhoea in as many as 58.7% of confirmed leptospirosis cases.

Another major challenge with the febrile illness is the co-infections with one or more pathogens which further complicates the diagnosis and thereby the clinical management. In the present study, we found seven leptospirosis patients to be co-infected with dengue and one with scrub typhus. Leptospirosis co-infection with dengue or scrub typhus is commonly seen in neighbouring countries such as Thailand and India [36–43]. Dengue is highly endemic in Malaysia with a minimum of 100,000 cases and more than 200 mortalities every year [44]. A recent study by Suppiah *et al*. (2017) reported 11/268 (4.1%) dengue cases to be co-infected with leptospirosis [45]. Although leptospirosis is endemic in Malaysia, the lack of clinical awareness and the evaluation of co-infection with dengue is often neglected, hence delays the appropriate management. It is difficult to differentiate between leptospirosis, dengue and scrub typhus solely based on clinical manifestations due to the overlapping signs and symptoms [46]. Thus, it is important to perform differential diagnosis among the three diseases not only based on the signs and symptoms but also through laboratory confirmation for effective management of the patients.

Molecular characterization based on 16S rDNA sequencing identified three *Leptospira* species (*L*. *interrogans*, *L*. *kirschneri*, and *L*. *wolffii*) to cause human leptospirosis in Central Malaysia. All three species have been previously isolated and identified from humans, animals or the environment in Malaysia. *L*. *kirschneri* and *L*. *interrogans*, the most common and widely distributed *Leptospira* species from rodents and small mammals [13,30, 47–51] were identified as the main human-infecting *Leptospira* species in Malaysia based on our study. *L*. *wolffii*, an intermediate species was detected in the blood of two patients. This species was initially isolated and described in human leptospirosis in Thailand [52] and has also been isolated from animals [53]. In Malaysia, earlier reports described the isolation of *L*. *wolffii* from soils and waters in residential areas of patients with leptospirosis [54], market and recreational areas [55]. This also further illustrates the contribution of *Leptospira* from the “intermediate” cluster as causes of human leptospirosis. We found a significant difference in the prevalence of *Leptospira* species identified in both states (Table 3), *L*. *kirschneri* was commonly found in Selangor, while *L*. *interrogans* largely predominated in Perak. However, reasons for the regional species domination is not clear and needs further research.

MLST is a gold standard typing method and useful for investigation of the evolutionary relationship between closely-related bacteria [56]. However, this method requires a high bacterial load (~5x10^4^ leptospires/ml). Consequently, success rates for obtaining partial and full profiles in clinical specimens were between 5% and 21% [57, 58]. An extended MLST based on nested PCR for typing of clinical samples was recently established by Weiss *et al*., and showed an improvement in obtaining full allelic profiles with 23% success rate [19]. Our present study utilized this extended MLST and from 63 samples tested, only one and eight *Leptospira* DNA from patients had full and partial allelic profiles respectively. The most frequently amplified loci were the *glmU* and *pntA*, which was also observed elsewhere [56]. The assignment of a new ST (ST 265) for the one sample that exhibited complete MLST profile implies that the locally distributed *Leptospira* strain is genetically distinct from those circulating internationally and in other geographic regions. These findings suggest the need to isolate the human-infecting strains in Malaysia to be included in the MAT panel to improve the diagnostic sensitivity. As reported by Mgode *et al*., the inclusion of local circulating serovars in the MAT panel could improve leptospirosis diagnosis [59]. In another study performed in Thailand, the leptospires serovars mostly found in patients were also the dominant serovars circulating in livestock [60]. In the present study, although similar species were identified among human and rodents [13], no link could be established at ST level. Based on the organized MLST allele pattern in Table 4, only ST 58 *L*. *interrogans* identified in human leptospirosis from Indonesia (neighbouring country) showed alleles for *glmU*, *pntA*, *sucA* and *caiB* similar to ST 265 *L*. *interrogans* observed in human samples in Malaysia. These data show that although similar species could be isolated from human and rodents, they are genetically different. Furthermore, *Leptospira* could be shed by other mammals like dogs, bats, livestock; hence, the reservoirs discovery deserves further research [61–65].

Our study has several strengths and limitations. We have identified *L*. *interrogans*, *L*. *kirschneri* and *L*. *wolffii* as the human-infecting *Leptospira* species in Malaysia. However, to conclude whether the same strain that is present in the rodent kidneys or in the environment is causing infection in human, it is crucial to identify the genotype to establish the epidemiological linkage. Although the MLST could be revealed for the *Leptospira* species isolated from rodents in our earlier study [13], the fact that no strains could be isolated from human samples or the DNA extracted from the blood samples was too little for performing PCR for all the seven alleles limits the epidemiological linkage understanding. Another major limitation of the study was the non-inclusion of urine samples to detect the late phase of illness as PCR is positive in blood only in the early phase and MAT in the immune phase (serum). The combination of both PCR and MAT for detection of leptospires in blood, serum and urine in both phases will be more robust and improve the diagnosis sensitivity. Hence, we recommend to perform a nationwide study to isolate and characterize the human-infecting strains from patients (blood and urine), continuous surveillance of animals (livestock, cats, dogs, bats) and the environment, as it is crucial to identify the genotype for determining the risk factors and effective management.

In summary, *L*. *interrogans*, *L*. *kirschneri*, and *L*. *wolffii* were identified as human-infecting species in Malaysia. ST 265 *L*. *interrogans* could be the major circulating genotype as 4/9 strains showed similar alleles for *glmU*, *pntA and mreA*, while others could be distinct STs. More studies on identifying the locally circulating *Leptospira* species from patients, animals and the environment are recommended to leverage our knowledge of the local epidemiology, for improving the diagnosis panel, hence assuring the effective management of the illness.

## Supporting information

S1 File Pro forma. The standardized interviewer administered questionnaire used for this study.
(PDF)

S2 File Supplementary Table 1. The sample IDs, species and GenBank accession number.
(DOC)
