# Supplementary material for: Leptospira interrogans and Leptospira kirschneri are the dominant Leptospira species causing human leptospirosis in Central Malaysia
Source: PLoS Negl Trop Dis. 2020 Mar 23;14(3):e0008197. doi: 10.1371/journal.pntd.0008197 (PMC7117766; doi:10.1371/journal.pntd.0008197)
Supplement: S1 File — The standardized interviewer administered questionnaire used for this study. (PDF) [file pntd.0008197.s001.pdf]

APPENDICES

<u>Pro forma</u>

Clinical profile of patients diagnosed with leptospirosis in
Hospital ___________________from _____________to________________

Case number:

Patient Registration Number:

Section 1: Demographic Data

1. Date of admission:
2. Age of admission:
3. Gender:
4. Ethnicity:
5. Department (admission wards):
6. Current address:
7. Height:
8. Weight:
9. Blood pressure:
10. Education level (Primary/Secondary/Tertiary):

11. Co-morbids (e.g diabetes, ischaemic heart disease, HIV etc). Please list patient's usual medication.

12. Is the patient on immunosuppresants?

13. Dieatary information (Vegatarian/ non-vegetarian)
14. Dietary supplements e.g zinc, calcium (Yes/No)
    A. If yes, please list

15. Smoking status (smoker / former smoker / never smoked)
16. Alcohol consumption (Yes/ Ex drinker/ No)

A. If yes	i:	Regular drinker (> 4 days a week)	( )
	ii:	Frequent drinker (1-4 days a week)	( )
	iii.	Occasional drinker (< 3 days a month)	( )

Section 2: Risk factors

1. Exposure to flood ( )
2. Involvement in an outdoor event (adventure race, triathlon) ( )
3. Involve in water activity (swimming, fishing, recreational) ( )
4. Involve with animals: name of the animals___________ ( )
5. Crowded housing/poor sanitation ( )
6. High risk occupation (gardener, sewage worker, vet etc) ( )

Section 3: Duration of illness at presentation

1. Day of illness at presentation________________ ( )
2. Visited a healthcare professional prior to presentation ( )
    A. If yes, was antibiotics prescribed?

Section 4: Clinical presentation

1. Asymptomatic ( )
2. Fever ( )
3. Headache ( )
5. Myalgia particularly associated with the calf muscles and lumber region ( )
6. Jaundice ( )
7. Conjunctival suffusion without purulent discharge ( )
8. Skin rash (maculopapular or petechial) ( )
9. Meningitis ( )
10. Gastrointestinal symptoms such as nausea, vomiting, abdominal pain, diarrhea ( )
11. Cardiac arrthythmia or failure ( )
12. ECG abnormalities ( )
13. Renal insufficiency (anuria or oliguria) ( )
14. Hemorrhages (from the intestines and lungs, hematuria, hematemesis ( )
15. Others:____________________________

Section 5: Markers for severe leptospirosis
Presence of at least one of these criteria:

1. Acute kidney injury ( )
    a. Serum creatine x 3 baseline or > 300 µmol
    b. Passing urine less than 0.3mls/kg/day or anuric > 12 hours
    c. Requiring renal replacement therapy
2. Severe hepatitis (alt > 1000 µmol/L) ( )
3. Severe jaundice (bilirubin > 70 µmol/L) ( )
4. Pulmonary hemorrhage ( )
    a. Frank hemoptysis or frank blood from endotracheal tube with at least two of the following:
        i. Drop of hemoglobin with no other explainable source of bleeding
        ii. No clinical signs of overload
        iii. Chest xray showing alveolar infiltration pattern
5. Mechanical ventilation ( )
6. Cardiovascular collapse requiring inotropic support ( )
7. Thrombocytopenia (< 70 000/ µL) ( )
8. Cardiac rhythm disorders (Atrial fibrilation, atrial flutter, heart blocks) ( )
9. Hemorrhagic manifestations ( )
10. Neurological involvement (facial palsies, altered GCS, meningeal syndrome ( )

Section 6: Laboratory diagnosis

Confirmatory

1. Leptospira agglutination titer of > 400 by MAT ( )
2. Detection of pathogenic Leptospira DNA by PCR ( )

Presumptive

1. Detection of IgM antibodies against Leptospira in acute serum specimen ( )

Section 7: Specimen (on admission/ on compilation/ on discharge

1. Types of specimen
   Blood ( )
   Respiratory ( )
   Urine ( )
   Others, specify :___________
2. Date of specimen :__________
3. Specimen code number:__________

Section 8: Other laboratory results

	Full Blood Count	Admission	Complications	Discharge	Normal Range
1.	WBC				4.0-11.0 x 10^9/L
2.	HB				14-18 g/dl
3.	Platelet count				150-450 x 10^9/L
4.	MCV				80-95 fL
5.	MCH				27-31 pg
6.	MCHC				32-36 g/dl
7.	Haematocrit				42-54%

Renal Profile

1.	Blood urea				5.0-7.0 mmol/L
2.	Creatinine				30-100 µmol/L
3.	Sodium (Na)				132-143 mmol/L
4.	Potassium (K)				3.2-5.7 mmol/L

Liver function test

1.	Total protein				60-80 mmol/L
2.	Albumin				35-50 g/l
3.	AST				10-50 U/L
4.	ALT				<50 U/L
5.	ALP				30-150 U/L
6.	Bilirubin				0.3-1.9 mg/dL
7.	ESR				< 20 mm/hr
8.	CRP				< 10 mg/dL
9.	Lactate				
10	Creatinine kinase				52-336 U/L

Arterial blood gas

1.	pH				
2.	pCO_2				
3.	pO_2				
4.	Bicarbonate				

Section 9: Relevant radiology investigation

1. Xray:
2. Ultrasound:
3. Ct Scan:
4. Others:

Section 10: Concomitant illness during hospital stay - with positive cultures

1. Pneumonia ( )
2. Cellulitis ( )
3. Bacterial meningitis ( )
4. Urinary tract infection ( )
5. Intrabdominal infection ( )
6. Dengue ( )
7. Malaria ( )

Section 11: Treatment for leptospirosis (antibiotics)

1. Name:
   Dosage:
   Duration:
   Started on:
   Stopped on:

2. Name:
   Dosage:
   Duration:
   Started on:
   Stopped on:

Section 12: Final diagnosis

1. Dengue ( )
2. Malaria ( )
3. Leptospirosis ( )
4. Melioidosis ( )
5. Bacteremia ( )
6. Unidentified acute febrile illness ( )
7. Others:___________

Section 13: Outcome

1. Survived ( )
2. Survived with complications ( )
   Please specify:______________________
3. Death ( )
4. Not sure ( )
