# Supplementary material for: Leptospira interrogans and Leptospira kirschneri are the dominant Leptospira species causing human leptospirosis in Central Malaysia
Source: PLoS Negl Trop Dis. 2020 Mar 23;14(3):e0008197. doi: 10.1371/journal.pntd.0008197 (PMC7117766; doi:10.1371/journal.pntd.0008197)
Supplement: S2 File — The sample IDs, species and GenBank accession number. (DOC) [file pntd.0008197.s002.doc]

| **Supplementary Table 1.** The sample IDs, species and GenBank accession number. | | | |
| --- | --- | --- | --- |
| **No** | **Sample ID** | **Species** | **GenBank Accession Number** |
|  | TI26 | *L. interrogans* | MK943577 |
|  | TI36 | *L. kirschneri* | MN006685 |
| 3. | TI42 | *L. interrogans* | MK943574 |
| 4. | TI48 | *L. interrogans* | MK943575 |
| 5. | TI51 | *L. interrogans* | MK943580 |
| 6. | TI55 | *L. interrogans* | MK943569 |
| 7. | TI58 | *L. interrogans* | MK943662 |
| 8. | TI66 | *L. interrogans* | MK943571 |
| 9. | TI68 | *L. interrogans* | MK943579 |
| 10. | TI72 | *L. interrogans* | MK943578 |
| 11. | TI73 | *L. interrogans* | MK943572 |
| 12. | TI76 | *L. interrogans* | MK943664 |
| 13. | TI88 | *L. interrogans* | MK943581 |
| 14. | TI90 | *L. interrogans* | MK943573 |
| 15. | TI94 | *L. interrogans* | MK943586 |
| 16. | TI95 | *L. interrogans* | MK943585 |
| 17. | TI101 | *L. interrogans* | MK943584 |
| 18. | TI103 | *L. kirschneri* | MK943671 |
| 19. | TI106 | *L. interrogans* | MK943661 |
| 20. | TI143 | *L. interrogans* | MK943672 |
| 21. | TI154 | *L. interrogans* | MK943669 |
| 22. | TI155 | *L. interrogans* | MK943668 |
| 23. | TI169 | *L. interrogans* | MN006687 |
| 24. | TI171 | *L. interrogans* | MK943583 |
| 25. | TI218 | *L. interrogans* | MK943673 |
| 26. | TI224 | *L. interrogans* | MK943588 |
| 27. | TI232 | *L. interrogans* | MK943587 |
| 28. | TI240 | *L. interrogans* | MK943663 |
| 29. | TI242 | *L. interrogans* | MK943667 |
| 30. | TI243 | *L. interrogans* | MK943660 |
| 31. | TI272 | *L. interrogans* | MK943658 |
| 32. | TI276 | *L. interrogans* | MK943665 |
| 33. | TI302 | *L. interrogans* | MK943659 |
| 34. | TI312 | *L. interrogans* | MK943670 |
| 35. | TI315 | *L. kirschneri* | MK943666 |
| 36. | SP3 | *L. kirschneri* | MK943566 |
| 37. | SP9 | *L. kirschneri* | MK943559 |
| 38. | SP10 | *L. kirschneri* | MK943549 |
| 39. | SP11 | *L. kirschneri* | MK943564 |
| 40. | SP14 | *L. kirschneri* | MK943567 |
| 41. | SP15 | *L. interrogans* | MK895089 |
| 42. | SP22 | *L. kirschneri* | MK943552 |
| 43. | SP23 | 1. *wolffii* | MN006690 |
| 44. | SP25 | *L. interrogans* | MK943554 |
| 45. | SP26 | *L. interrogans* | MK943550 |
| 46. | SP27 | *L. interrogans* | MK943556 |
| 47. | SP28 | *L. kirschneri* | MK943555 |
| 48. | SP29 | *L. interrogans* | MK943557 |
| 49. | SP30 | *L. interrogans* | MK943553 |
| 50. | SP32 | *L. interrogans* | MK943568 |
| 51. | SP33 | *L. kirschneri* | MK943560 |
| 52. | SP39 | *L. interrogans* | MN007188 |
| 53. | SP44 | *L. kirschneri* | MK943563 |
| 54. | SP45 | *L. kirschneri* | MK943551 |
| 55. | SP47 | *L. kirschneri* | MK943558 |
| 56. | SP57 | *L. kirschneri* | MK942438 |
| 57. | HT61 | *L. interrogans* | MK943582 |
| 58. | HT65 | *L. wolffii* | MN007192 |
| 59. | HT66 | *L. interrogans* | MK943576 |
| 60. | HT69 | *L. interrogans* | MK943570 |
| 61. | HT71 | *L. interrogans* | MK943562 |
| 62. | HT72 | *L. kirschneri* | MK943561 |
| 63. | HT73 | *L. kirschneri* | MK943565 |
